# The McKenzie Method Is an Effective Rehabilitation Paradigm for Treating Adults With Moderate-to-Severe Neck Pain: A Systematic Review With Meta-Analysis

**DOI:** 10.7759/cureus.39218

**Published:** 2023-05-19

**Authors:** Anthony N Baumann, Kevin Orellana, Leah Landis, Marc Crawford, Caleb J Oleson, Hudson Rogers, Deven P Curtis, Keith D Baldwin

**Affiliations:** 1 Department of Rehabilitation Services, University Hospitals, Cleveland, USA; 2 Department of Orthopedics, Children's Hospital of Philadelphia, Philadelphia, USA; 3 Department of Rehabilitation and Sports Therapy, Cleveland Clinic Akron General, Akron, USA; 4 Department of Physical Therapy, Stability Physical Therapy, Ashtabula, USA; 5 Department of Medicine, Northeast Ohio Medical University, Rootstown, USA

**Keywords:** cervicalgia, neck pain, physical therapy, exercise, mckenzie

## Abstract

Neck pain is a common musculoskeletal condition frequently managed with numerous conservative interventions. The McKenzie method of mechanical diagnosis and therapy (MMDT) is a form of physical therapy evaluation and treatment that aims to improve pain and disability in patients with musculoskeletal pain, including neck pain. To date, no systematic review with meta-analysis has examined the use of the McKenzie MMDT for neck pain. This study aimed to examine the effectiveness of the McKenzie MMDT in adult patients with neck pain. A systematic review and meta-analysis were performed using PubMed, ScienceDirect, MEDLINE, CINAHL, Web of Science, and Google Scholar. Full search terms were “McKenzie method” OR “McKenzie approach” OR “McKenzie treatment” AND “neck pain.” Inclusion criteria were the use of the McKenzie MMDT, level I randomized control trials (RCTs), adults, and outcomes of pain (0-10 scale) and disability (neck disability index). A total of 11 RCTs met the final selection criteria from 1,955 articles on initial search with 289 patients receiving the McKenzie MMDT out of 677 total patients. For meta-analysis, there was a clinically insignificant but statistically significant improvement in pain (1.14/10 points) in patients receiving the McKenzie MMDT versus control interventions (p<0.02). There was no significant improvement in the neck disability index score between the McKenzie MMDT versus control interventions (p=0.19). For severity of pain, there was a clinically and statistically significant improvement in moderate or severe pain (2.06/10 points; p<0.01), but not in mild-to-moderate pain (p=0.84) when comparing the McKenzie MMDT to control interventions. Overall, the McKenzie MMDT provides very small but statistically significant improvements in neck pain of all severity compared to control interventions. However, the McKenzie MMDT does provide clinically and statistically significant pain improvement in moderate-to-severe neck pain. Use of the McKenzie MMDT did not provide any significant improvement in disability compared to control interventions. This study is the first systematic review with meta-analysis on the effectiveness of the McKenzie MMDT for adult patients with neck pain.

## Introduction and background

Neck pain is a common musculoskeletal condition often treated conservatively in various ways in the outpatient setting [1-3]. An estimated 70% of people will have neck pain at some point in their lives with 54% of individuals having neck pain within the last six months [1,4]. Furthermore, the risk of neck pain recurrence and chronicity is high with up to 5% of adults being disabled due to neck pain [1,3]. Neck pain can be managed by many interventions and paradigms within physical therapy as physical therapy is considered to be one of the main conservative treatment options for neck pain [1,3]. It is estimated that up to 25% of outpatient physical therapy treatments are allocated towards patients with cervical spine issues, highlighting the high economic burden associated with neck pain in adults [1].

The McKenzie method of mechanical diagnosis and therapy (MMDT) is a commonly used approach for the management of spinal and extremity pain, including neck pain [4-8]. The treatment-based approach of the McKenzie MMDT is dependent on a thorough history, physical examination, and response to repeated motions [4]. In the McKenzie MMDT, patients are placed into subcategories based on examination findings from repeated spinal motions and treated accordingly with the goal of maximizing patient self-management of symptoms [4]. While extensive research has been done on the effectiveness of the McKenzie MMDT on low back pain, limited data exist on the effectiveness of the McKenzie MMDT for patients with neck pain [9,10].

The most recent systematic review on the effectiveness of the McKenzie MMDT for the cervical spine was completed almost one decade ago, included five trials, and was unable to perform a meta-analysis due to heterogeneity of study outcomes [4]. The overall conclusion of that systematic review was that the added benefit of the McKenzie MMDT compared to control or wait-and-see approaches may not be clinically important in terms of pain or disability [4]. Since that time, multiple high-quality studies examining the effectiveness of the McKenzie MMDT have been completed, prompting a new systematic review and first-time meta-analysis [7,8,11-15]. The purpose of this systematic review and meta-analysis is to examine the effectiveness of the McKenzie MMDT for adult patients with non-specific neck pain to improve evidence-based care and patient outcomes.

## Review

Methods

Initial Search

A systematic review was performed using PubMed, ScienceDirect, MEDLINE, CINAHL, Web of Science, and Google Scholar from December 5, 2022, to database inception. Full search terms included “McKenzie method” OR “McKenzie approach” OR “McKenzie treatment” AND “neck pain” in each database. No filters were used to perform the initial search.

Inclusion and Exclusion Criteria

Inclusion criteria were patients receiving physical therapy treatment, usage of the McKenzie MMDT in adult patients, pain in the cervical region only, any subtype of cervical pain, and records of pain outcomes (0-10 scale including visual analog scale and the numerical pain rating scale) and disability outcomes (neck disability index {NDI}). Only level I randomized controlled trials (RCTs) were included in this systematic review. Exclusion criteria were no interventions using the McKenzie MMDT, pain outcomes not on a 0-10 scale, disability outcomes other than the NDI, pediatric patients, and thoracic pain concurrent with cervical pain. Studies that were level of evidence II or below were excluded including but not limited to case reports, case series, systematic reviews, and meta-analyses. Articles were also excluded if they were only abstracts, were not accessible despite multiple attempts, and did not have full text in English.

Article Screening, Data Extraction, and Bias and Quality Assessment

Multiple authors participated in title and article screening, full-text screening, and data selection. Any discrepancies were decided by the first author. The author’s believed that the vast number of articles on Google Scholar was important to the creation of this systematic review and meta-analysis. Due to the limitations of Google Scholar, each article had to be manually downloaded in order to be included in this systematic review. The initial Google Scholar search yielded 1,240 articles; however, there was a lack of access to all articles beyond page 49 on the database, with each page containing 20 articles. This “service error” was consistent across multiple devices and browsers. Therefore, we were only able to retrieve the first 980 articles from Google Scholar. The authors reached out to the engineers at Google Scholar but received no reply after several months. All included articles were assessed with the Physiotherapy Evidence Database (PEDro) scale for bias and quality assessment. 

Meta-Analysis

A meta-analysis with a random effects continuous model was constructed to compare continuous outcomes between studies for patients who received the McKenzie MMDT or control interventions (conventional physiotherapy, isometric exercises, or other exercises/interventions). The primary analysis for our study question was a random effects analysis of RCTs comparing pain and NDI change in patients who received the McKenzie MMDT compared to control interventions. Forest plots were generated and used to assess for study heterogeneity and to provide summary estimates. In accordance with previous reviews with study heterogeneity (I^2^>0.75), a random effects model was chosen to combine the effects of multiple studies [16,17]. The unstandardized mean difference was used to analyze the post-treatment effects of the McKenzie MMDT in studies with different control and treatment groups. A p-value less than 0.05 was considered statistically significant. Meta-analysis was done using SPSS statistical software version 28.0 (Armonk, NY: IBM Corp). Frequency-weighted means were used to generate average follow-up times.

Results

Study Selection

**Figure 1:**
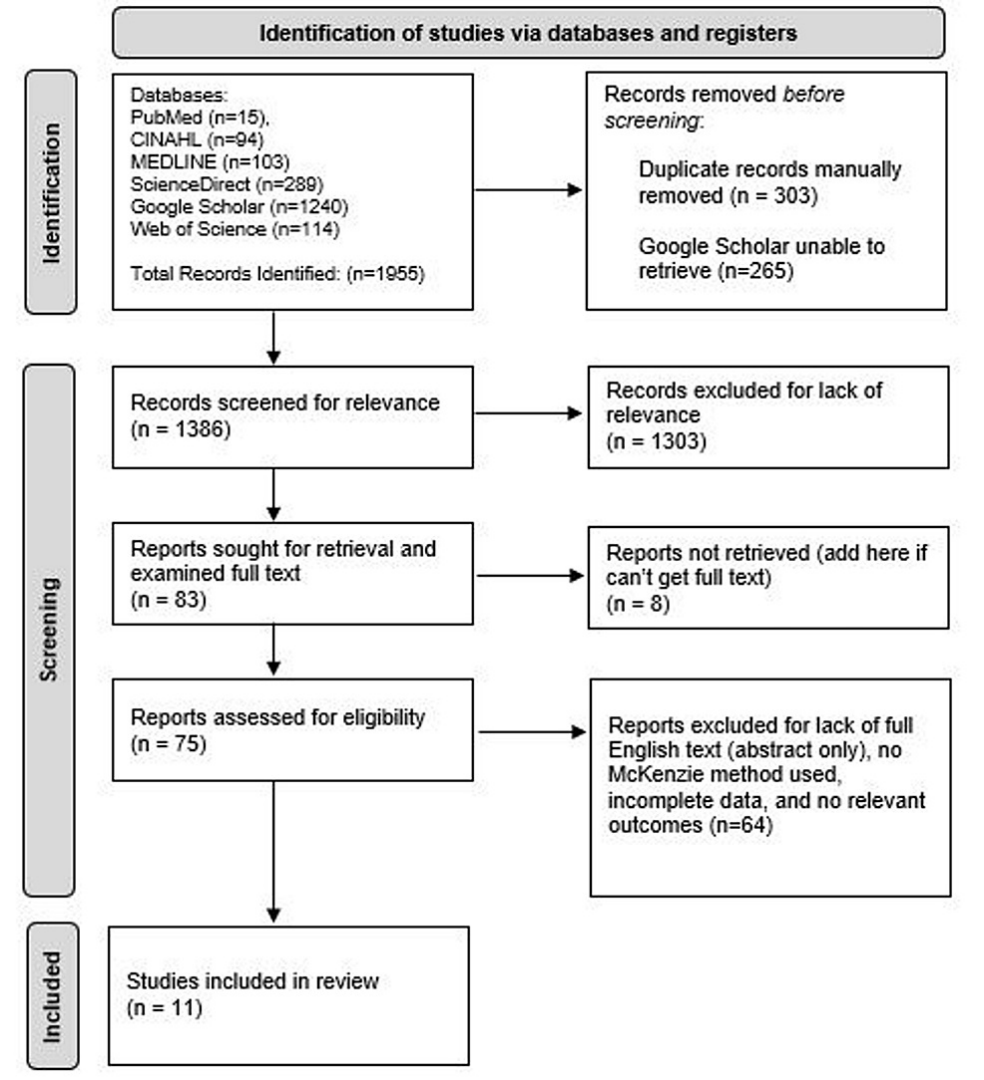
PRISMA diagram for this systematic review describing the initial search terms, title and abstract screening, full-text screening, and final studies included in this review. PRISMA: Preferred Reporting Items for Systematic Reviews and Meta-Analysis

A total of 1,955 articles were retrieved from PubMed, ScienceDirect, MEDLINE, CINAHL, Web of Science, and Google Scholar databases on December 5, 2022, with 11 RCTs meeting the final selection criteria for the current systematic review and meta-analysis [7,8,11-15,18-21]. Figure 1 and Table 1 show the Preferred Reporting Items for Systematic Reviews and Meta-Analyses (PRISMA) diagram and bias/quality assessment, respectively. A total of 677 adult patients were examined within the 11 RCTs with a total of 289 patients receiving the McKenzie MMDT for neck pain. The average follow-up time for all studies was 13.6±10.2 weeks. Ten studies examined the effect each treatment arm had on neck pain levels (n=647 total patients) with 269 patients receiving the McKenzie MMDT, while five studies examined the effect each treatment arm had on disability via the NDI (n=201 total patients) with 98 patients receiving the McKenzie MMDT. Table 2 represents the information regarding all articles in the present study with data points for NDI, pain scores, and follow-up times.

**Table 1:** The quality of articles and risk of bias were assessed using the PEDro scale. The assessment was completed by multiple authors. The PEDro scale has 10 graded criteria (0-1 points) for a total of 10 possible points. PEDro: Physiotherapy Evidence Database

Author (year)	PEDro Score	Eligibility criteria were specified	Random allocation	Allocation was concealed	Groups similar at baseline	Subject blinding	Therapist blinding	Assessor blinding	Less than 15% dropouts	Intention-to-treat analysis	Between-group statistical comparisons	Point measures and variability data
Kumar (2010) [18]	6	Yes	1	0	1	0	0	0	1	1	1	1
Arshad et al. (2020) [11]	7	Yes	1	1	1	0	0	0	1	1	1	1
Miecznikowski et al. (2019) [12]	5	Yes	1	1	1	0	0	0	0	0	1	1
Seo et al. (2012) [19]	7	Yes	1	1	1	0	0	0	1	1	1	1
Lee et al. (2017) [13]	7	Yes	1	1	1	0	0	0	1	1	1	1
Kotagiri et al. (2018) [14]	6	Yes	1	1	1	0	0	0	0	1	1	1
Elmeged et al. (2021) [8]	7	Yes	1	1	1	0	0	0	1	1	1	1
Law (2015) [20]	8	Yes	1	1	1	0	1	0	1	1	1	1
Yana et al. (2021) [15]	4	Yes	1	0	1	0	0	0	0	0	1	1
Ammar (2018) [21]	6	Yes	1	1	1	0	0	0	1	0	1	1
Abdel-Aziem et al. (2022) [7]	5	Yes	1	1	1	0	0	0	1	0	0	1

**Table 2:** Summary of all articles included in this study. RCT: randomized controlled trial; VAS: visual analog score; NDI: neck disability index

Author (year)	Study type	Patients (n)	Group	Pre-VAS pain	Post-VAS pain	Pre-NDI score	Post-NDI score	Follow-up (weeks)
Kumar (2010) [18]	RCT	10	Experimental: conventional plus McKenzie	8.1 (1.7)	0.7 (0.5)	-	-	1.4
		10	Control: conventional plus neural mobilization	6.5 (2.0)	1.9 (1.3)	-	-	1.4
		10	Control: conventional (short wave diathermy, intermittent cervical traction)	7.8 (2.4)	1.0 (0.7)	-	-	1.4
Arshad et al. (2020) [11]	RCT	20	Control: isometric exercise	5.8 (0.4)	3.8 (0.7)	-	-	4
		20	Experimental: McKenzie treatment	6.1 (0.6)	3.0 (0.7)	-	-	4
Miecznikowski et al. (2019) [12]	RCT	42	Experimental: McKenzie	4.2 (0.6)	1.9 (0.8)	13.8 (1.9)	9.9 (2.1)	6
		44	Control: suboccipital relaxation	4.3 (0.6)	1.9 (0.7)	14.7 (1.9)	11.3 (2.0)	6
Seo et al. (2012) [19]	RCT	10	Control: sling exercise	3.2 (1.4)	1.1 (1.0)	7.2 (2.0)	4.2 (1.4)	4
		8	Experimental: McKenzie exercise	3.3 (1.5)	1.9 (1.5)	7.3 (1.3)	5.3 (2.3)	4
Lee et al. (2017) [13]	RCT	11	Experimental: app-based McKenzie exercises	5.2 (2.2)	2.7 (2.0)	-	-	8
		9	Control: brochure on exercise	4.0 (1.8)	3.7 (2.0)	-	-	8
Kotagiri et al. (2018) [14]	RCT	30	Control: Mulligan mobilization with upper limb movement	7.4 (1.0)	4.0 (1.0)	-	-	4
		30	Experimental: McKenzie exercises with neural mobilization	7.1 (0.7)	2.0 (0.6)	-	-	4
Elmeged et al. (2021) [8]	RCT	15	Control: conventional physiotherapy	8.6 (0.7)	8.0 (0.8)	30.1 (3.9)	30.1 (3.8)	8
		15	Experimental: conventional physiotherapy with McKenzie exercises	8.0 (0.9)	3.3 (1.0)	30.8 (7.0)	18.3 (7.8)	8
Law (2015) [20]	RCT	84	Control: home exercise and spinal care workshop	2.1 (2.2)	1.3 (1.8)	-	-	26
		84	Core stabilization	1.8 (2.3)	0.8 (1.6)	-	-	26
		103	Neck McKenzie exercise	1.5 (2.0)	0.6 (1.4)	-	-	26
Yana et al. (2021) [15]	RCT	20	Group A: deep neck flexor strengthening exercises, postural advice	-	-	41.2 (4.0)	35.0 (3.8)	6
		20	Group B: McKenzie neck exercises with postural advice	-	-	40.9 (4.8)	36.6 (4.7)	6
Ammar (2018) [21]	RCT	14	Stabilization group	4.3 (1.6)	1.4 (0.7)	7.2 (1.3)	4.0 (1.2)	6
		13	McKenzie exercise group	4.4 (1.4)	1.8 (0.8)	7.2 (1.5)	2.5 (2.4)	6
Abdel-Aziem et al. (2022) [7]	RCT	20	Control: traditional therapy	6.2 (1.1)	4.6 (1.1)	-	-	6
		18	Control: deep neck flexor strengthening and exercise	6.3 (1.8)	3.5 (1.2)	-	-	6
		17	Experimental: McKenzie group	6.5 (1.8)	2.4 (1.0)	-	-	6

McKenzie Versus Control Interventions Outcomes

**Figure 2:**
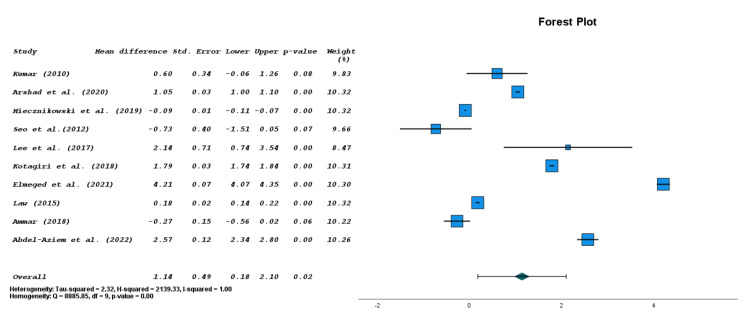
Forest plot for McKenzie MDT versus control for pain levels. MDT: mechanical diagnosis and therapy

Weighted mean pain level for the control group was 3.60/10 before treatment and 1.80/10 after treatment. Weighted mean pain level for the McKenzie MMDT group was 3.85/10 before treatment and 1.85/10 following treatment. Using meta-analysis to compare post-treatment pain level differences between McKenzie MDT and control groups in the 10 RCT studies comparing pre- and post-treatment pain revealed a small but statistically significant mean pain improvement of 1.14 points with McKenzie MDT (CI=0.18, 2.10; p<0.02) (Figure 2) [7,8,11-14,18-21]. However, this mean pain improvement falls below the minimal clinically important difference (MCID) of 1.37/10 points for the pain scale as recorded for other orthopedic conditions [22]. For studies examining pain between the McKenzie MMDT and controls (n=637), the average follow-up was 14.1±10.4 weeks. When comparing post-treatment NDI difference in the five studies comparing the McKenzie MMDT to control therapy, a 2.09/100 point improvement was seen; however, it was not statistically or clinically significant (CI= -1.06, 5.25; p=0.19). Five studies focused specifically on how pain levels differed between the McKenzie MMDT and conventional physiotherapy groups. When analyzing the post-treatment pain levels following treatment, the McKenzie MMDT had a statistically insignificant 1.56-point pain improvement when compared to conventional physical therapy alone (CI= -0.10, 3.23; p=0.07). However, this improvement of pain with McKenzie MDT was approaching statistical significance in comparison to improvement of pain with conventional physical therapy (p=0.07) and represents a clinically significant decrease in pain [22].

Mild-to-Moderate Versus Moderate-to-Severe Pain Levels

**Figure 3:**
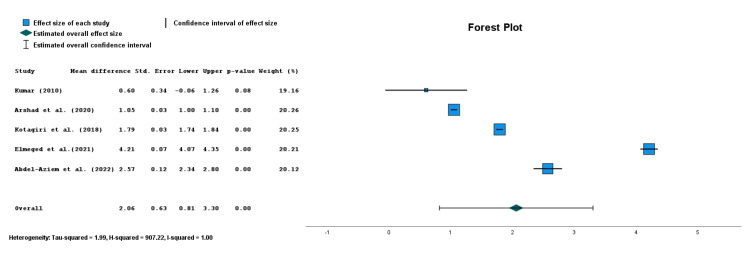
Forest plot for McKenzie MDT impact on pain improvement in studies with moderate-to-severe mean pain levels. MDT: mechanical diagnosis and therapy

The authors were particularly interested in seeing the effect that the use of the McKenzie MMDT had on patients with both mild-to-moderate or moderate-to-severe pain. The 10 RCTs comparing post-treatment pain outcomes were divided based on a reported mean pain level equal to or greater than 5/10. Patients with pain beneath the threshold were classified as having mild-to-moderate pain, while patients with pain levels of five or greater were classified as having moderate-to-severe pain. Five studies with a mean patient pain level of less than five were included. When comparing post-treatment pain differences between the McKenzie MMDT and control groups in patients with mild-to-moderate neck pain, there was a non-significant mean pain level improvement of 0.06 points (CI=-0.56, 0.68; p=0.84). However, when comparing post-treatment pain in the five studies with moderate-to-severe pain, a statistically and clinically significant difference of 2.06 points was seen with the use of McKenzie MDT as compared to improvement from the control interventions (CI=0.81, 3.30; p=0.001) (Figure 3) [7,8,11,14,18].

Discussion

This study is the first combined systematic review and meta-analysis on the effectiveness of the McKenzie MMDT in adult patients with neck pain. Since the last attempt at systematic review in 2014 on the effectiveness of the McKenzie MMDT for neck pain, numerous high-quality RCTs have been completed, including nine of the 11 studies included in this review [4,7,8,11-15,20,21]. Based on the results of this study, the McKenzie MMDT can provide small but statistically significant improvements in pain, but not disability, in adult patients with neck pain as compared to control interventions. However, this small improvement falls below the MCID for pain and thus represents a clinically insignificant improvement in pain when applied to all patients with neck pain regardless of pain severity. Furthermore, this study was able to demonstrate that McKenzie MMDT is more effective in patients with higher levels of neck pain (greater or equal to 5/10) compared to controls, whereas the superiority of the McKenzie MMDT disappeared in patients with mild neck pain (less than 5/10). Therefore, this study can only recommend the McKenzie MMDT as superior to control interventions for adult patients with moderate-to-severe neck pain.

The literature suggests that the superiority of the McKenzie MMDT depends on the type of control interventions used for comparison in the treatment of low back pain [10]. Similar limitations may exist with neck pain, as there are a wide variety of treatments commonly used for the conservative management of neck pain [1,9,10]. Control groups compared to the McKenzie MMDT in this study included neural mobilization, intermittent cervical traction, isometric exercise, suboccipital relaxation, sling exercise, exercise brochures, conventional physical therapy, deep neck flexor strengthening exercises, and stabilization exercises. However, the authors believe that it is important to compare the McKenzie MMDT to the many other interventions used by clinicians for neck pain in order to demonstrate if the McKenzie MMDT is truly superior to other interventions.

When compared to conventional physical therapy alone, the McKenzie MMDT did not produce statistically significant improvements in pain. However, this may be due to the relatively small sample size compared directly to conventional physical therapy, as the results were trending toward statistical significance. It should be noted that the pain improvement for the McKenzie MMDT versus conventional physical therapy was higher than the MCID of 1.37/10 points on the pain scale, thus indicating clinically significant improvements in pain [22]. While a recent systematic review and meta-analysis were able to examine the effectiveness of the McKenzie MMDT on low back pain depending on acuity, this study was not able to comment on the utility of the McKenzie MMDT based on symptom duration [10].

This study has several limitations that impact the recommendations and generalizability of the results. One limitation is that the usage of the McKenzie MMDT can vary in quality between practitioners in the clinic and in clinical trials. Furthermore, it is possible that differing levels of training in the McKenzie MMDT can impact effectiveness. Unfortunately, the McKenzie MMDT is sometimes incorrectly equated with simple extension spinal exercises, which could possibly limit effectiveness and hide the true utility of the McKenzie MMDT as an approach to spinal pain [23]. Therefore, these results should be interpreted with caution as an ideal application of the McKenzie MMDT can be dependent on many factors. More research is needed to determine the impact of the McKenzie MMDT based on clinician training and certification. Another limitation is the scope of the McKenzie MMDT itself, as the McKenzie MMDT represents a vast array of diagnoses, treatments, and an approach to spinal pain [5]. As the McKenzie MMDT is an approach, rather than a simple technique, there are inherent limitations in comparing individual utilization of the McKenzie MMDT. As the literature supports multimodal physical therapy treatments, there may be value in adding the McKenzie MMDT to a more comprehensive treatment approach rather than in isolation [1,2]. The McKenzie MMDT offers an approach that can treat many different spinal syndromes, such as neck pain with radiating pain; however, this study only examined patients who exclusively had non-specific neck pain and was not specific for neck pain primarily due to stiffness or cervical radiculopathy [1,2]. The effectiveness of the McKenzie MMDT could vary depending on the different subsets of neck pain; however, the findings of this study are not able to comment on this limitation. Finally, more high-quality RCTs are needed on this topic as several of the studies included in this systematic review and meta-analysis were of lower quality, thus impacting the strength of the recommendation of this study. Of note, this study is only able to comment on the short-term impact of the McKenzie MMDT for neck pain as many of the included studies had short follow-up times. More research is needed to determine the impact of the McKenzie MMDT on patients with neck pain based on acuity, the impact of clinician training on neck pain outcomes, and the overall utilization of the McKenzie MMDT on different subsets of neck pain.

## Conclusions

The use of the McKenzie MMDT provides clinically insignificant but statistically significant improvements in neck pain as compared to control interventions for adults with neck pain regardless of pain severity. However, the McKenzie MMDT did produce clinically and statistically significant improvements in neck pain in adult patients with moderate-to-severe neck pain as compared to control interventions. The McKenzie MMDT did not produce significant improvement in pain levels compared to control interventions in adult patients with mild-to-moderate neck pain. Furthermore, the use of the McKenzie MMDT did not provide any significant improvement in disability compared to control interventions. When compared to conventional physical therapy alone, the McKenzie MMDT did not provide any significant improvement in pain levels but was trending towards statistical significance. However, these results were limited by a relatively small sample size. This study is the first systematic review and meta-analysis of the effectiveness of the McKenzie MMDT for adult patients with neck pain.

In conclusion, the use of the McKenzie MMDT method can be cautiously recommended for patients with moderate-to-severe neck pain, while more extensive research is still needed to determine the impact of McKenzie MDT on neck pain in adult patients.
